# First results of the Maastricht brace in the treatment of adolescent idiopathic scoliosis

**DOI:** 10.1186/1748-7161-9-S1-O34

**Published:** 2014-12-04

**Authors:** Dirk Schrander, Joris Hermus, Helma Voets, Mark van den Boogaart, Paul Willems, Lodewijk van Rhijn

**Affiliations:** 1 Maastricht Universitair Medisch Centrum, Maastricht, the Netherlands

## Background

The Maastricht brace (M-brace) was developed to improve compliance and associated efficacy of brace treatment in adolescent idiopathic scoliosis (AIS). Initial pressure measurements in the M-brace revealed a higher corrective pressure as compared to the Boston brace, and a better patient reported quality of life, as measured with the SRS 22 and Brace questionnaire. We present the first results of the efficacy in terms of curve correction of the M-brace in AIS.

## Aim

The aim of this study was to evaluate the in-brace curve correction of the Maastricht brace and to determine the effect of increased wearing comfort on treatment efficacy.

## Design

Retrospective cohort study.

## Methods

A total of 46 patients (mean age of 13 years) with mild to moderate AIS, who have been treated with the M-brace since January 2011, were included. The correction effectiveness of the brace was evaluated by comparing the primary and secondary curves on bending x-rays with those on standard postero-anterior full spine radiographs with and without M-brace. The degree of correction in the M- brace was then expressed as a percentage of the correction as achieved in the bending radiographs. As a control group four patients were also fitted a Boston brace, in order to compare the in-brace correction between the braces.

## Results

There were 38 patients with a primary thoracic curve, and 8 patients with a primary lumbar curve. The average primary curve angle measured in Cobb degrees was 34.7° ± 11.3°. The average primary curve angle in bending x-rays was 15.5° ± 8.3°. In the M-brace the primary curve was 25.4° ± 10.1° (p<0.01). This is an in-brace correction of 48%. The control group had an in-brace correction of 49.7% in the Boston brace versus 45.1% in the M-brace (p=0.21).

## Conclusions

These preliminary results demonstrate an adequate in-brace correction of the M-brace, which is comparable to corrections found in current literature and similar to the in-brace correction of the Boston brace in the control group. Given the relationship between compliance and wearing comfort, the M-brace is, without compromising treatment efficacy, a promising new brace treatment for adolescent idiopathic scoliosis.

